# Acoustic Description of the Soundscape of a Real-Life Intensive Farm and Its Impact on Animal Welfare: A Preliminary Analysis of Farm Sounds and Bird Vocalisations

**DOI:** 10.3390/s20174732

**Published:** 2020-08-21

**Authors:** Gerardo José Ginovart-Panisello, Rosa Ma Alsina-Pagès, Ignasi Iriondo Sanz, Tesa Panisello Monjo, Marcel Call Prat

**Affiliations:** 1 Grup de Recerca en Tecnologies Mèdia (GTM), La Salle—Universitat Ramon Llull, C/Quatre Camins, 30, 08022 Barcelona, Spain; gerardojose.ginovart@salle.url.edu; 2 Cealvet SLu, C/Sant Josep de la Montanya 50-B, 43500 Tortosa, Spain; tesapm@cealvet.com; 3 Grup de Recerca en Technology Enhanced Learning (GRETEL), La Salle—Universitat Ramon Llull, C/Quatre Camins, 30, 08022 Barcelona, Spain; ignasi.iriondo@salle.url.edu; 4 Bonarea Agrupa, C/ Transpalau n°8, 25210 Guissona, Spain; marcel.call@bonarea.com

**Keywords:** *Leq*, farm management noise, bird well-fare, stress, vocalisation frequency, poultry farm, weight, food and water intake

## Abstract

Poultry meat is the world’s primary source of animal protein due to low cost and is widely eaten at a global level. However, intensive production is required to supply the demand although it generates stress to animals and welfare problems, which have to be reduced or eradicated for the better health of birds. In this study, bird welfare is measured by certain indicators: CO_2_, temperature, humidity, weight, deaths, food, and water intake. Additionally, we approach an acoustic analysis of bird vocalisations as a possible metric to add to the aforementioned parameters. For this purpose, an acoustic recording and analysis of an entire production cycle of an intensive broiler Ross 308 poultry farm in the Mediterranean area was performed. The acoustic dataset generated was processed to obtain the Equivalent Level ($L_{eq}$), the mean Peak Frequency (PF), and the PF variation, every 30 min. This acoustical analysis aims to evaluate the relation between traditional indicators (death, weight, and CO_2_) as well as acoustical metrics (equivalent level impact ($L_{eq}$) and Peak Frequency) of a complete intensive production cycle. As a result, relation between CO_2_ and humidity versus $L_{eq}$ was found, as well as decreases in vocalisation when the intake of food and water was large.

## 1. Introduction

In recent years, genetic selection has been performed over the years to increase the growth rate in the shortest possible time [1] in the context of the poultry meat industry [2]. The demand for poultry food due for its low price and nutritional properties, projects a continuous expansion of the poultry market [3]. This demand for white meat has increasingly led to genetic selection for a fast early growth rate that may provoke the appearance of several spontaneous, idiopathic muscle abnormalities along with an increased susceptibility to stress-induced myopathy [4] in modern chick strains. Causes of mortality related to fast growth are mainly Sudden Death Syndrome [5] and ascites [6]. Nevertheless intensive production is also a source of stress for animals. Some of these factors such as stocking density, environmental deterioration, unsuitable social environments, and thermal stress can be major sources of stress [7]. Moreover routine management practices are stressful for birds [8,9]. An important management practice is the ventilation of poultry houses, as this could influence the gas emissions of birds and the subsequent intensive production of, for example, CO_2_, CH_4_, or N_2_O [10]. Carbon dioxide production CO_2_ is used in poultry as a gas meter to determine the ventilation flow of the farm according to the International Commission of Agricultural Engineering [11]. Ventilation renews gas concentrations by reducing pollutant gas levels and increasing the amount of oxygen on the farm. For instance, a low value of CO_2_ indicates good ventilation and is a sign of good animal welfare. All such technologies that support a closer attention to the animals, not only for better welfare and health but also for sustainability, are included in the Precision Livestock Farming (PLF) concept. For more details about these new trends that prioritise more attention to animals rather than only watching the numbers, an extensive review by Norton et al. can be found in [12].

The welfare of animals has become an important fact for society in many countries of the world. This fact, together with the automatising of most animal monitoring processes, can support the farmer in the care of animals. Following this idea, bioacoustics studies the biological significance and the characteristics of sounds emitted by living organisms [13], and can be a relevant issue to complement the traditional measurements (CO_2_, temperature, etc.) of environmental characteristics in a farm. Threat signals [14], information about feeding [15], or sexual selection [16] are only some examples of the possible applications of this field. More details about the acoustic analysis in the framework of farm management and more precisely, about the acoustic analysis of birds vocalisations are given in the related work.

More particularly, the field of birds is one of the few groups of animals known to exhibit vocal learning, used for communication for territoriality, high density, food/water restriction, heat-cold stress, alarm signalling, among others [17]. The bird song is recorded using a non-invasive method, with the aim of analysing their song and correlating data. Several indicators about bird vocalisations have already been reported with dependencies in literature with birds weight, as a conclusion of their welfare [18]. In this study, we design and analyse the recording campaign of an entire production cycle in a Mediterranean farm during the winter season to obtain acoustic data. This acoustical analysis aims to evaluate the relation between traditional indicators (death, weight, and CO_2_), acoustical metrics (equivalent level impact ($L_{eq}$), Peak Frequency (PF)), and farm management information (food and water intake, temperature, and humidity) of a complete intensive production cycle of around 40,000 Ross 308. All acoustic data recorded will later be processed and analysed, considering other metrics of the farm management. This work is the preliminary analysis for the correlation between all these available parameters about a farm environment and management, and bird growth and vocalisations. In order to model bird welfare in intensive production farms, the wider the available information about the life of the animals, the more accurate the dependencies may be found.

This paper is structured as follows. The related work used as framework of this project is detailed in Section 2. The specification of the farm where the project has been implemented is detailed in Section 3. The acoustical analysis of the recording can be found in Section 3.3. Bird welfare data analysis is detailed in Section 4. Discussion of the key aspects of this work is detailed in Section 5 and the conclusion as well as future work can be found in Section 6.

## 2. Related Work

In recent years, the welfare of farm animals has become an important issue for societies in many countries of the world. Automating animal monitoring processes, such as acoustic analysis of their vocalisations, can greatly assist farmers in this type of task. Therefore, it is important to review the acoustic analysis of commercial chicken farming.

### 2.1. Acoustic Analysis of Farm Management

In nature, the vocal sounds produced by different animal species are related to certain functions, such as threat signals (alarm calls to different predators [14]), information about feeding (food-associated calls [15]), or sexual selection [16]. In many species, these sounds can reveal attributes related to the caller’s identity, sex, age, reproductive status, or social dominance [19]. Therefore, vocalisation, the active generation of sounds with specific organs, becomes an expression of an internal state of an animal generated spontaneously or motivated by an external event [20]. Many of these vocalisations have a complex structure that includes different acoustic elements and there are many hypotheses related to the adaptive function of how such complexity [21] have developed over years. The study of emotions in animals is related to the evolution of species and consequently to the evolution of animal vocalisations. In terms of arousal, it is likely that vocal correlations with negative mood states such as alarm calls or infant begging calls, emerged earlier during evolution than positive vocalisations. For more information, the reader is referred to [22], which presents a review of the current state of knowledge on vocal correlations of emotions in humans and other mammals.

In recent years, animal welfare has become a very important issue for the scientific community and general public. This generalised demand for greater respect for animals covers multiple areas such as the treatment of domestic animals or those that are kept in zoos, but this request becomes more relevant in all aspects related to farm-raised animals [23,24]. As a consequence, administrations have adopted a series of recommendations and directives to protect farm animals [25], although regulations promoted for each country are directly related to the level of public concern for the welfare of farmed animals. Social demands often influence the programs of political parties and therefore the action of governments. For example, this pressure is much higher in countries like the UK and Germany than others like Spain or Italy [26]. In any case, new initiatives are emerging around supranational organisations that group public and private institutions like the Welfare Quality^®^ Network (www.welfarequality.net), which define four animal welfare principles: Good housing, good feeding, good health, and appropriate behaviour.

Bioacoustics, which is the study of animal sound communication, is performed in farm environments by using recorders capable of automatically recording audio data [27]. Animal welfare monitoring can be substantially improved through an increased use of automated methods and, therefore, one promising area in particular is the use of automated analysis of animal vocalisations. A first step to improving animal welfare is to maintain animals free of pain, injury, or disease. In [28], a literature review includes different types of indicators that allow pain assessment in some mammals, birds, and fish. Vocalisations are included in a set of behavioural indicators along with posture, isolation, lack of appetite, or others. This study concludes that these indicators have the best chances of detecting pain early with a combination of them or even just one. For instance, in main farm mammals (pigs, cattle, or lambs), there are changes in the number and duration of vocalisations, intensity, and spectral characteristics. These kind of vocalisation changes are also observed in hens during the removal of feathers or picking. Other state-of-the-art studies centred in vocalisation of different farm animal species can be found in [20,27]. In this kind of research, it is essential to identify screams due to pain or stressful situations from other sounds [29] and also to know the vocal behaviour of farm animals (cattle [30], pigs [29], and chickens [18]). One of the main current trends in this research field is heading towards the development of farm animal vocalisation classification algorithms, combining different audio parameters with automatic classification systems [31].

### 2.2. Acoustic Analysis of Bird Vocalisations for Welfare Evaluation

Among the different farm animals, our research is addressed to acoustic analysis in poultry farms. Therefore, we start from the study of information that relates their vocalisations and their relation with welfare. Fontana et al. [18] present a complete study of the young bird vocalisations in an attempt to find some patterns depending on the age (1 day or 5 days of life) and the situation of the chickens (isolated or in group). They found 12 different frequency patterns concluding that the type of vocalisations changes from “call sounds” to “distress calls” as the birds grew. Furthermore, audio samples (spectrograms) of chicken vocalisations have been used to distinguish healthy from infected (infectious bronchitis) birds [32]. Carpentier et al. [33] presents an algorithm to monitor chicken sneezing sounds assuming an environment where there are several noise sources and multiple birds vocalisations. Another issue to take into account is the highly unbalanced nature of the raw acoustic dataset. The algorithm is designed to support in the diagnose of poultry health in farms, especially focused on respiratory diseases, which are a major health problem.

Lee et al. [34] use more acoustic parameters to automatically detect stress in laying hens. Abdel-Kafy et al. [35] found a highly significant negative correlation between the peak frequency of vocalisations and the weight and age of turkeys. Du et al. [36] also address stress in laying hens by means of their vocalisation analysis, with the final goal of assessing their thermal comfort condition. They apply a nine source-filter structure to both temporal and spectral features, and a Support Vector Machine to classify the different animal responses.

De Moura et al. [37] presented a study that correlates the environmental temperature with the behaviour and vocalisation of chicks. They detected changes in the intensity and frequency of their vocalisations when temperature decreases. In this case, chicks try to warm up by gathering and in order to reduce the heat loss of the flock. There are other important sounds apart from vocalisations such as pecking that can be used to monitor the food intake of the chickens [38] by placing a microphone in the feeder instead of a device attached to each animal. This is a key point to achieve a non- invasive system capable of continuous audio measurements.

In a recent work, Herborn et al. [17] present a single acoustic marker that co-varies with a range of physical, behavioural, and emotional welfare concerns. This marker, called by the authors as iceberg indicator is the spectral entropy measured after the clean low frequency sound of machinery. With this acoustic parameter, they showed a linear correlation with the manual distress call count in the first 4 days of placement and therefore were able to predict low weight gain and high mortality for the following days.

In our opinion, there are some interesting approaches that include the use of sound analysis on commercial chicken farming, but there is still a long way to go to achieve a complete and robust system that helps farmers to improve the welfare of chicks. This statement is in line with the conclusions of the review presented by Rowe et al. [39]. They analyse the degree of development of the Precision Livestock Farming (PLF) technology in poultry farming. They conclude that the main goal of PLF development is improving animal welfare over increasing production, although the availability of commercial systems available to farmers is still scarce. With respect to the sensors used in poultry PLF, they found that cameras were used in a large proportion of the studies (42.42%) while the use of microphones was less popular (14.02%). Another review, comprising 57 studies, found that only 8% used sound technology [40]. Therefore, the general trend in PLF is the capture of a lot of data from different kind of sensors that must be processed with big data and internet of things technologies to facilitate the smart management of poultry [41].

## 3. Materials and Methods

Automated chicken farms allow the continuous monitoring and measurement of the environment affecting poultry production. In this study a farm with the following technical specifications was chosen in order to be able to contrast and compare the data with the metrics of the acoustic animal vocalisations.

### 3.1. Environment

The acoustic analysis was performed in a Mediterranean farm of the BonArea Agrupa corporation (www.bonarea-agrupa.com) of 42,840 commercial chicken farming during an entire Ross 308 production cycle [42], which represents a total of 44 days of life. The study was held in the winter season last January to the beginning of March 2020. The average temperature in the outer farm was between 6 and 15 °C, the humidity close to 0% and a rainfall average of 10% (Meteorological data obtained from on 25 May 2020, https://es.climate-data.org/europe/espana/cataluna/sidamon-662610/).

**Figure 1:**
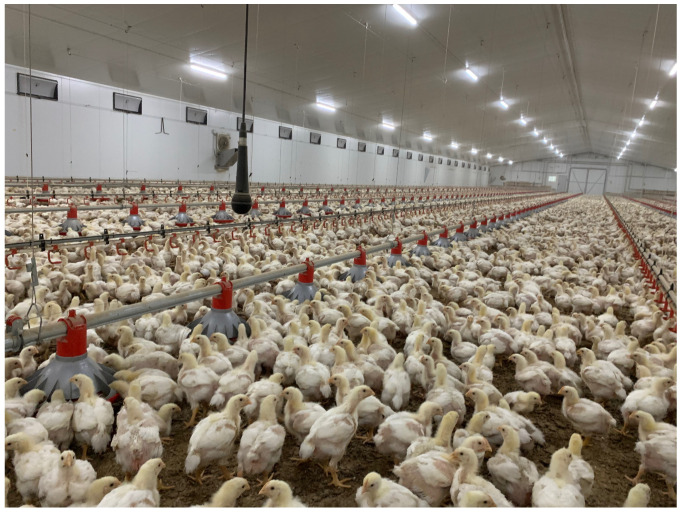
Picture of day 21 in H1 house. The birds live with the microphone installation. It is recording continuous raw acoustic data.

The farm chosen has almost two identical chicken houses of 20 m × 120 m total size each (see Figure 1), both of which are fully instrumented with the following machinery: (i) Underfloor heating, with hot water production by use of propane gas, (ii) an additional heating system with hot air generators, (iii) forced ventilation by tunnel system, and (iv) a heat exchanger installed in one of the buildings. There is also a sensor network that records CO_2_ levels, and the humidity, as well as the inner and outer temperature. The network sensors and some manual rules introduced to the system by the farmer automatise the farm management in terms on activation of ventilation, heating, and light. Food and water supply are also automatised and guaranteed throughout all the production cycle for all birds by means of refilling the containers when the food is scarce. The characteristics of this farm provides a suitable environment for this study. The automation reduces the human factor in farm management and provides data of the environment and productivity factors that can be analysed together with animal vocalisation metrics.

In order to certify the equivalence of the measurements, the sensors were identically installed in each animal house to collect raw data in order to provide redundancy of data, in case one of the measurements presents problems during the recording campaign. One farm was analysed (H1) with a backup for any inconvenience of (H2). The vocalisations of the chickens were recorded throughout the cycle, in order to evaluate the background equivalent level $L_{eq}$ [43] and the frequencies of the vocalisations and their dependencies with other environmental measurements.

### 3.2. Materials

The goal of the recording campaign was to collect both vocalisations and background noise of commercial chicken farming throughout their life-cycle, in order to evaluate the evolution of the entire production time for further analysis. The vocalisations captured by the microphone are group vocalisations due to the animal density and sensor location. For this reason, single identifications could not be performed. Nevertheless, the purpose of this work is to evaluate the entire animals’ welfare, not individual bird tracking.

**Figure 2:**
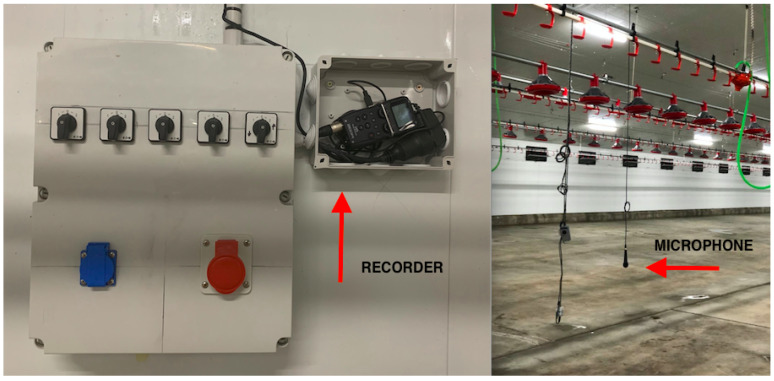
Acoustic equipment deployment in the farm H1. On the left, the recorder location. On the right, the microphone hanging from the ceiling in order to avoid physical interaction with the birds.

A professional handheld recorder (Zoom H5) [44] was used, connected to a directional microphone Behringer ultravoice XM1800S with a frequency response of 80–15 kHz and a sensibility of 2.5 mV/Pa [45]. The sounds emitted by birds in each house were recorded with one microphone each, deployed one meter high from the ground and at the centre to the house. Figure 2 shows the acoustic sensor deployment. The location was chosen to avoid chickens interfering with the microphone (biting, singing just next to it, etc.) and also to provide a wide background of sound recordings. The microphone diagram pattern was selected in order to reduce maximum interference of other source sounds, such as machinery due to its cardiod shape. Similar acoustic implementation techniques have been used in other studies [46,47].

The Zoom H5 handheld recorder was configured to record the entire production cycle with as few data stops as possible. Although the recorder stopped when it reached the 32 Gb of data due to the maximum continuous recording storage, in this project setup it takes approximately 6 days to stop. To ensure continuous audio recording after 5 days, the system was stopped for a periodical technical reset. The data was collected from the SD to a hard disk and after a small stop of approximately 15 min the system was reactivated. By default these 5 days were stored in audio pieces of 6.75 h duration for further processing. The recording format was PCM-16 and the sampling rate was set to 44.1 kHz. The post processing analysis required a time reference of each measure to obtain reliable results especially when comparing with other data collected in the farm. For that purpose, each audio was saved with the metadata of the storing time of the file. By the end of the project, the 44 days of chicken vocalisations generated around 400 Gb of data describing the events and welfare of the chickens on the farm.

**Figure 3:**
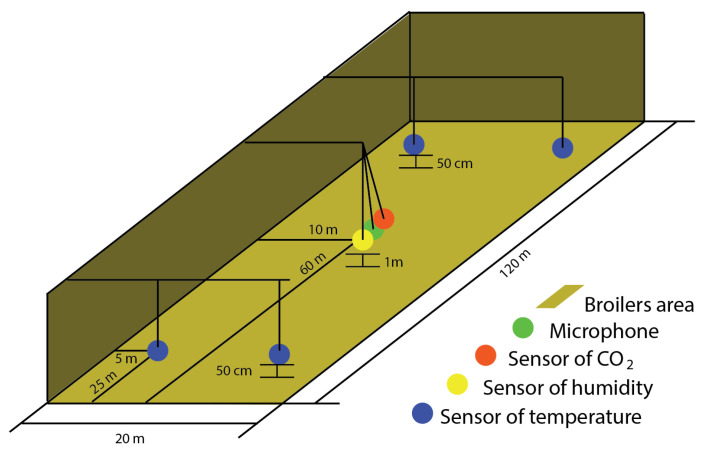
Diagram of the sensors location in the H1 farm, microphone, CO_2_ sensor, humidity, and temperature sensor.

The selected farm has a work dynamic where CO_2_, temperature, humidity, losses, and weight are measured in each animal production cycle. These 5 data variables were provided by the farm. CO_2_, temperature and humidity measurements were carried out every 15 min, the mortality of animals was obtained daily, and the average weight weekly. The CO_2_, temperature, and humidity network sensors (see Figure 3) were distributed through the room and all data were collected in a hard disk via a management software for the daily management of the farm.

The animals’ weight and mortality were manually obtained. Birds’ weight evaluation has to be representative from all the chicken house. The calculation method uses an electronic scales to weigh N=100 animals and calculate the mean value (see Equation (1)):

(1) $$Weight_{mean}=\frac{1}{N}\sum_{i=1}^{N}BirdsW_{i}$$

Significant results have to use at least N=100 animals or 1% of the population [48]. For each calculation the digital scale was calibrated and birds were sampled from at least 3 different points of the house. The frequency of weight calculation during the cycle was set weekly as important weights variances were found in periodicity.

Part of the farmer’s daily routine is to check around the farm early in the morning. Daily farm inspection enables the farmer to detect possible diseases, supply chain problems, any birds problems, and find and remove dead chickens, which reduces gases generated of the birds decomposition. The farmer documents the number of deaths and the statistically average weight of the animals as data for each production cycle. This information is supplied by the farmer to this study.

### 3.3. Methods

Recording birds songs is a non-invasive method, that can measure animal acoustic parameters and relate them for example with welfare without modifying their natural behaviour as done in other studies [7,8,9]. In this study, we want to find a dependency between the acoustic characteristics and the usual indicators of the farm.

#### 3.3.1. Acoustic Metrics Defined to Measure the Raw Acoustic Data

After the production cycle, 44 days of raw acoustic data were obtained. The system was reset 9 times during the entire project and a total amount of 160 files were saved. Each file takes up 2.15 Gb, configured as mono channel sampled at 44.1 kHz and 16 bits has a duration of 6 h 45 min and 46 s. From these 160 files, there are 9 files that were manually reset and therefore have a variable length due to the time of the technician’s operation.

The system presented a failure on the 10th and 11th days due to a technical issue other than the known technical stops used to reset the hardware. All the usable data were processed using MATLAB^®^ [49].

##### $L_{eq}$  in the Farm

The acoustic equivalent level $L_{eq}$ is defined as a value of the sound pressure level of a continuous, steady sound that, within a specified time interval, has the same mean square sound pressure as a sound under consideration whose level varies with time [50,51]. As Equation (2) states, it is a logarithmic measurement:

(2) $$L_{eq}=10log\left(\frac{1}{T}\int_{0}^{T}\frac{P_{i}{\left(t\right)}^{2}}{P_{ref}^{2}}dt\right)$$

In this study the time interval chosen for the $L_{eq}$ is 30 min. This interval has been decided in order to obtain the same resolution as the two CO_2_ samples, as well as for computational reasons in this stage of the project. These acoustic feature indicates the intensity of the sound averaged in 30 min according to a sound pressure of reference. As most of the recorded and analysed sounds are birds vocalisations, it depicts the intensity animals singing.

The microphone was not been calibrated for this project for the following reasons: (i) the handheld recorder is designed for audio recording not as a measurement instrumentation, (ii) the recorder can not fine-tune the sensibility of the microphone, and (iii) the cardioid microphone used is a commercial voice microphone, not a Class 1 microphone, as those microphones have an unidirectional pattern non desired for the project requirements. Likewise the $L_{eq}$ measurements in this study aim to evaluate equivalent level variations, not requiring a high accuracy measurement as in a Class 1 device.

##### Peak Frequency During the Recording Campaign

There are almost 12 different chicken vocalisations identified in the literature that have a different spectral pattern [18]. Statistical analysis showed a significant correlation (*p* < 0.001) between the frequency of vocalisation and the age of the birds [18]. Birds peak frequencies vocalisation range between 2.7–4.3 kHz. According to the results of this study, it was found that the main frequency of the sounds emitted by birds is inversely proportional to their age and weight, specifically, the more they grew, the lower the frequency of the sounds made by the birds.

In the present study, the spectral bandwidth acquired is limited by the recorder configuration to 22.05 kHz, due to the sampling frequency at 44.10 kHz. To avoid interference of other sounds sources (machinery, people talking, etc.), raw audio data is filtered using a bandpass filter with a response of 2 to 5 kHz, reducing potential interference noise at frequencies other than those generated by animals.

**Figure 4:**
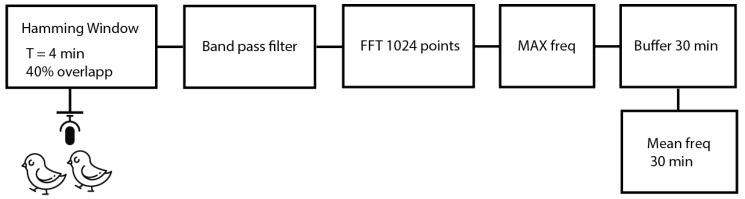
Peak frequency detection algorithm implementation.

To obtain the peak frequency, the following algorithm is applied (see the equivalent block diagram in Figure 4):

1. Data is segmented using Hamming windows of 4 min [52] and overlap of 40% between consecutive windows;
2. Data is filtered using a band pass filter from 2 to 5 kHz;
3. A FFT (Fast Fourier Transform) algorithm of 1024 points is applied [53];
4. The maximum value of the window is extracted;
5. Buffering of 30 min;
6. Calculate the mean peak frequency of the 30 min.

##### Identification of Machinery Sound Data

The acoustical data acquisition method has been specifically designed to capture the birds vocalisations. Unfortunately, some sounds of the fan, feeders, and several bar vibration of the feeders are also recorded. The microphone position (vertical to the ground) and its cardioid pattern (available on datasheet [45]) reduce the influence of acoustic events that do not correspond to vocalisations of the closest animals [54].

**Figure 5:**
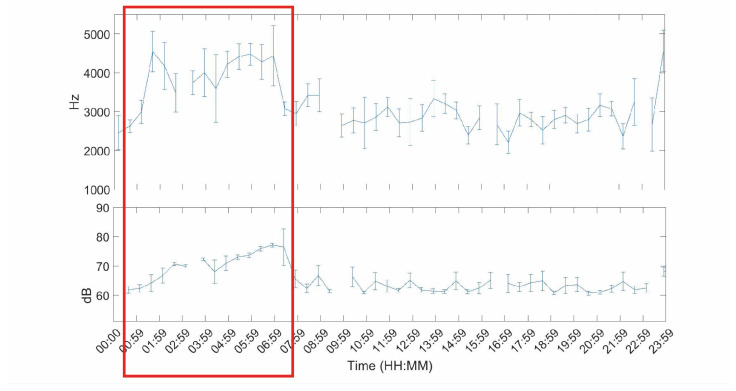
Temporal $L_{eq}$ sample in which the increase due to machinery is clearly identificable. In the horizontal axis we find the time and in the vertical axis the frequency (**top**) and the $L_{eq}$ (**bottom**).

This non desired captured events are easy to identify and also to exclude from the analysis. Figure 5 shows a sample of average $L_{eq}$ values over 24 h. In this sequence, the machinery sound datum is identified as the sound that stand out for a high and long-lasting equivalent level. The non desired event is highlighted in red and corresponds to the sound of airborne feed in the supply chain.

The acoustic profile is studied in more detail in terms of $L_{eq}$ and frequency variations. The maximum frequency is found between 4–4.5 kHz with variations of more than 1 kHz. Meanwhile, terms of $L_{eq}$ the range corresponds from 60 to 80 dB with small variations (±2.5 dB).

#### 3.3.2. Evaluation of the Acoustic Raw Data

**Figure 6:**
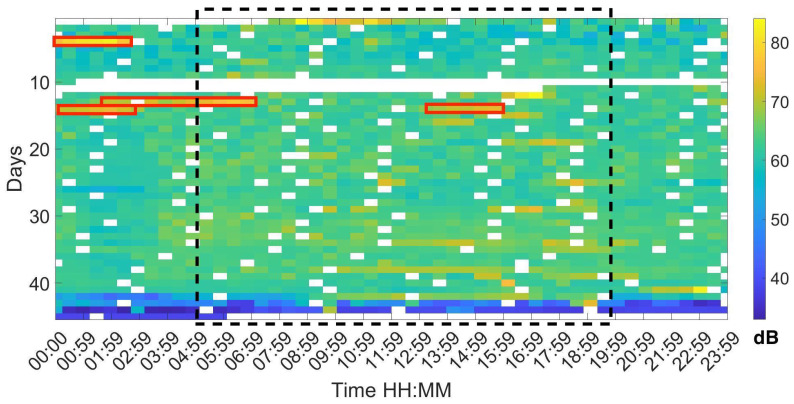
Map of the $L_{eq}$ at 30 min intervals during the 44 days of a complete production cycle. The red rectangles correspond to the areas where there is a high $L_{eq}$ measurement due to machinery.

**Figure 7:**
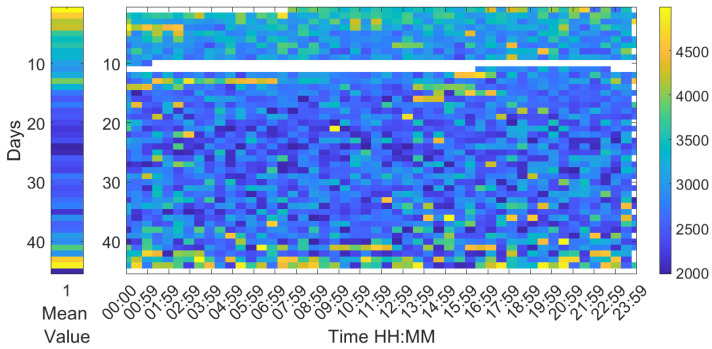
Map of the maximum frequency at 30 min intervals during the 44 days of a complete production cycle. The horizontal axis shows the hours of the day and night, and the vertical axis shows the days of the cycle.

**Figure 8:**
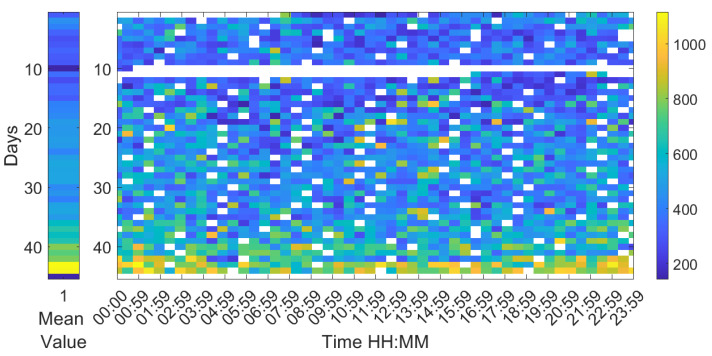
Map of the variance in frequency at 30 min intervals during the 44 days of a complete production cycle. The horizontal axis shows the hours of the day and night, and the vertical axis shows the days of the cycle.

Analysing the $L_{eq30\min}$ and maximum frequency each 30 min over the entire production cycle show the evolution of the sound pressure, and highest frequency generated by the birds according to their life expectancy. Figure 6, Figure 7 and Figure 8 reflect this study. The white cells representing data are missing files, that could not been computed due to the hardware limitation of the processing unit of the acquisition system.

Figure 6 shows the sound pressure evolution generated by the birds in a complete production cycle. There were 42,840 animals until day 33, when the density of animals is reduced. Therefore, during the last days of their life cycle, there were less chickens in the house and as a consequence, sound pressure was reduced.

As shown in Figure 6 there is no age-related increase in the level of pressure of chickens as the mean level is not increased with time. From five in the morning until nine in the evening coinciding with the period of more activity we can appreciate an increase in $L_{eq}$ of more than 7 dB. The temporal area with more activity is highlighted in a black discontinued rectangle. We highlighted with red rectangles some periods that present a high acoustic level due to the machinery identification. A careful analysis of these segments, louder and clearer vocalisations can be heard from the chickens closest to the microphone.

However, Figure 7 firmly shows an age-related decrease in peak frequency throughout the whole production cycle. Otherwise there is not a relevant variation on a daily basis. The frequency obtained on the first and last day of life of animals is higher with respect to the average values of those days. High-stress moments reflect an increase in the frequency of vocalisation in the data.

Apart from the mean value of the peak frequency, it is also relevant to measure its variance with the further intention of detecting possible correlations with other parameters being evaluated. Each 30 min segment of data has been processed in 4 min windows and the variance of the peak frequency has been calculated (see Figure 8). In general, an age-related increase is observed, as well as an increase during the night with respect to the day. However, picking up the birds at the end of production shows the highest frequency variations of all samples.

## 4. Experiments and Results

This section describes the traditional data farm indicators of a production cycle: Temperature, humidity, weight, CO_2_, food, and water intake. All traditional data were obtained on a regular basis as indicators that help the farmer during the production cycle. These data were provided by the farmer.

This study analyses the acoustical data with the farm management data: $L_{eq}$ and max frequency, with the traditional data. Some relevant relations of this two blocks of data that have been found in this analysis are: (i) Correlation between the maximum frequency of vocalisation versus food and water intake, (ii) CO_2_ versus $L_{eq}$, and (iii) humidity versus $L_{eq}$. Direct relations between variables within the same group have also been identified. This sections detail all relevant similarities found in the cross-data study.

### 4.1. Farm Management Data

Data shown in this section: CO_2_, temperature, humidity, weight, deaths count, food, and water intake has been provided by the farm manager and extracted from the farm’s automated control system. Traditional data values indicates a good production cycle to be analysed and studied as a standard uncomplicated breeding.

**Figure 9:**
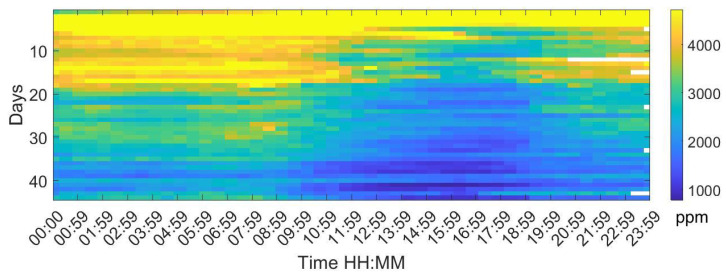
Map of the mean CO_2_ values for each day of the campaign. The horizontal axis shows the hours of the day and night, displaying a value every 30 min, and the vertical axis shows the days of the cycle.

Figure 9 shows the evolution of the CO_2_. Carbon dioxide CO_2_ is exhaled by the chickens, the release of manure, and the gas-fired combustion. An increase in this gas is observed when the manure is moved.

A high concentration of CO_2_ at the beginning of breeding corresponds to the need to maintain an indoor temperature of 32 °C during the first 5 days of life of the chickens and 30 °C between 5 to 10 days, so the ventilation rate should be low in order to optimise the indoor temperature, an effect that is more pronounced in colder months.

Higher concentrations of CO_2_ are detected as from day 10 from eight in the evening to ten in the morning reducing the gas concentration to 3000 ppm due to the ventilation. Day 20 of life onward show the highest reduction. Ventilation patterns reduce the concentration of gases. The manure movements are performed during the morning by the farmer and also reflect the increase of gas concentration in that time slot.

**Figure 10:**
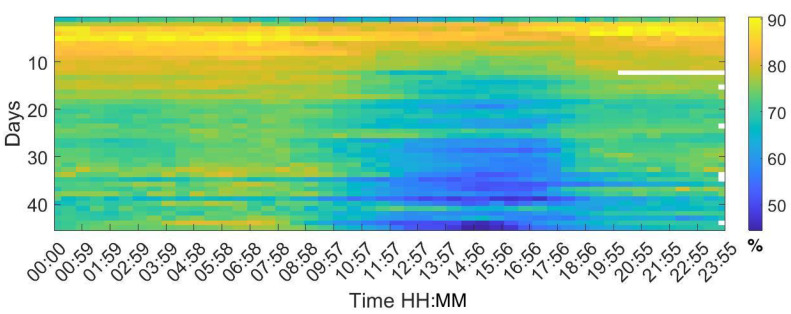
Map of the humidity values for each day of the campaign. The horizontal axis shows the hours of the day and night, displaying a value every 30 min, and the vertical axis shows the days of the cycle.

Similar patterns can be observed with the humidity in Figure 10. The highest values are recorded in the first week and it is continuous during the entire day. From day 10 onward a decrease of more than 10% is found between 10 to 18 h, evolving the window of humidity the last days of the cycle with two more hours of lower humidity measurement.

**Figure 11:**
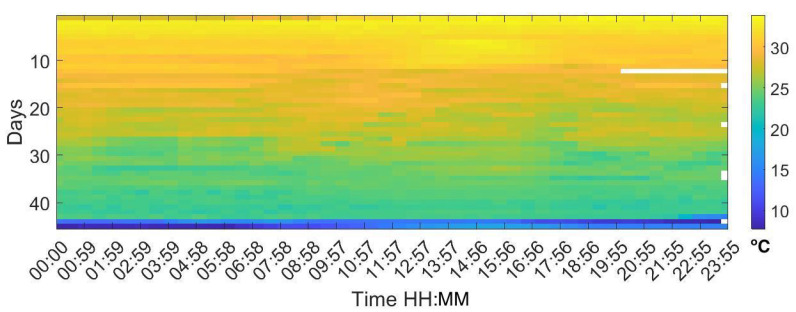
Map of the temperature values for each day of the campaign. The horizontal axis shows the hours of the day and night, displaying a value every 30 min, and the vertical axis shows the days of the cycle.

Otherwise, temperature has a different pattern shown in Figure 11. Young birds have little ability to regulate their internal temperature and they need heat, at a temperature of approximately 32 °C at their first week of life and the farm provides it externally. Temperature onward is slowly reduced until day 25 when a peak in temperature is reached (from seven to eight). Since day 30, temperature is lowered and homogeneous during the rest of the day.

**Figure 12:**
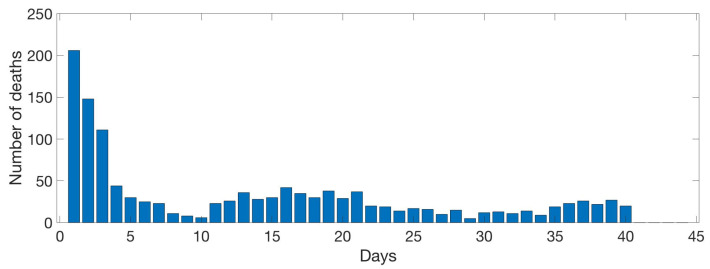
Evolution of the animal death count per day. Data evaluated daily by the farm management.

Animal death count is shown in Figure 12, where in the first week birds have the highest mortality by premature death, although it decreases in an almost exponential manner. Starting the second week, the number of deaths per day is sporadic. From the second week and onwards two more local maximums are found in day 17 and 37.

Animal weight average measurements are shown in Table 1. Birds weights are variant between animals, the average weight values represents the total of animals. The mean value is calculated using 100 birds. This process requires time and is only performed once per week.

**Figure 13:**
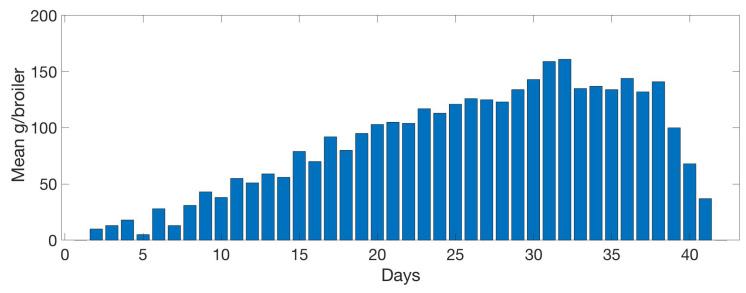
Evolution of the mean food intake per day by the birds. Data collected daily by the farm management.

Figure 13 shows the mean food intake per bird each day. Reduction of the intake is found in the last 3 days due to the manual reduction of animals in a farm, which is not reflected in the system. A linear growth behaviour can be observed until day 31 when maximum food production is reached, food consumption, obtained a peak value of around 150 g. From day 33 to 38 food intake stabilised to 140 g.

**Figure 14:**
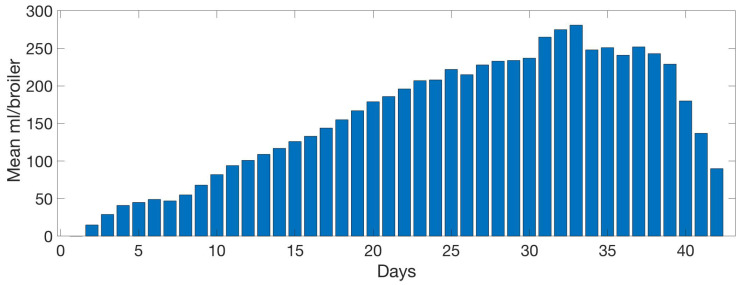
Evolution of the mean water intake per day by the birds. Data collected daily by the farm management.

A similar pattern can be observed in Figure 14. The graph shows the mean water intake per bird each day. The last 3 days reflect the animal reduction as seen in Figure 13. The growing linear model lasts until day 33 with the maximum bird water intake in day 33, to days later compared with food intake in Figure 13. Then the water consumption stabilised to 230 mL until day 39.

### 4.2. Evaluation of the Correlation between Acoustic Data and Welfare Information

Circular correlation is calculated as [55] describes. Let y(k) and x(k) be N-point signals, and let $x_{p}\left(k\right)$ be the periodic extension of x(k). The circular cross-correlation of y(k) with x(k) is denoted $c_{yx}\left(k\right)$ and defined in Equation (3):

(3) $$c_{yx}\left(k\right)\overset{\Delta }{=}\frac{1}{N}\sum_{i=0}^{N-1}y\left(i\right)x_{p}\left(i-k\right),0\leq k<N$$

This study computed all the correlations between traditional and acoustical data. Significant results are shown from Figure 15, Figure 16, Figure 17, Figure 18, Figure 19, Figure 20, Figure 21 and Figure 22. And a detailed list of the non clear correlation is also provided.

**Figure 15:**
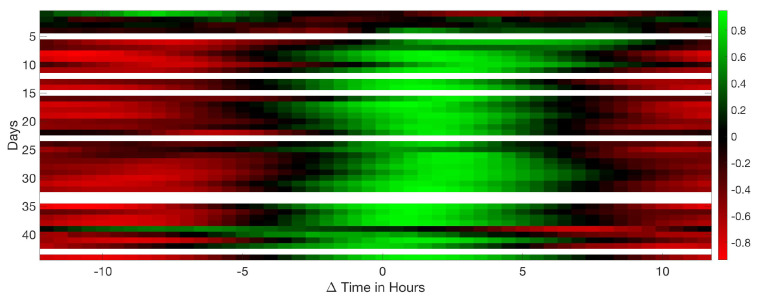
Results of the circular correlation CO_2_—humidity. Horizontal axis corresponds to the ΔTime measured in hours, evaluating the delay between CO_2_ and humidity. Vertical axis stands for the days of the cycle.

In Figure 15 we observe a clear correlation between CO_2_ and humidity, and the maximum values for all the days fall nearly in the centre of the circular correlation, which leads us to infer that they are two measured parameters in the farm that present similarities in their performance. This means that when the levels of the CO_2_ are greater, so is the humidity. A certain time delay was recorded on a number of days, this variation of maximum 5 h, where the humidity is delayed in its performance in comparison with CO_2_. Carbon dioxide is produced by the exhalation of the animals, so the greater the exhalation larger the contribution of humidity. When the ventilation is switched on, the CO_2_ and the humidity are reduced in the building.

**Figure 16:**
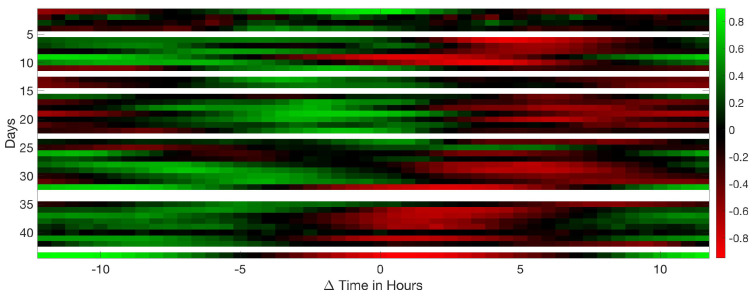
Results of the circular correlation CO_2_—temperature. Horizontal axis corresponds to the ΔTime measured in hours, evaluating the delay between CO_2_ and temperature. Vertical axis stands for the days of the cycle.

In Figure 16 we can observe a correlation between CO_2_ and temperature, in this case there is an inverse dependency. CO_2_ is in advance of the temperature, when CO_2_ increases the value, in a delay between 5 and 10 h, the temperature decreases. Outer temperature is considerably low, henceforth air flow injected to the farm is cold. After ventilation is reduced and the CO_2_ falls, its CO_2_ value after a few hours the temperature rises again.

**Figure 17:**
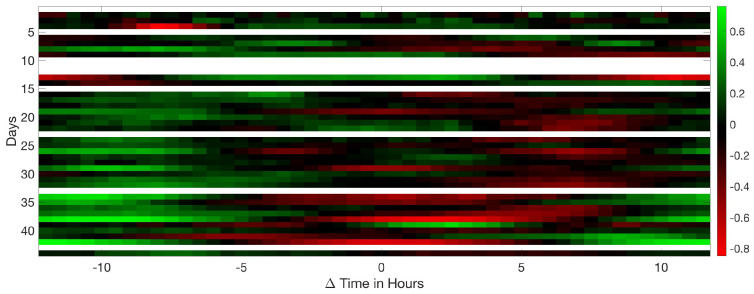
Results of the circular correlation CO_2_—$L_{eq}$. Horizontal axis corresponds to the ΔTime measured in hours, evaluating the delay between CO_2_ and $L_{eq}$. Vertical axis stands for the days of the cycle.

Figure 17 show a slight inverse similarity of the CO_2_ referenced to the equivalent level $L_{eq}$, with a different performance for the entire production cycle. When CO_2_ is at a maximum, the sound of birds vocalisation is minimum and in reverse. More vocalisation is an indicator of bird activity and increases the $L_{eq}$. Therefore when the CO_2_ is reduced, the vocal activity increases.

**Figure 18:**
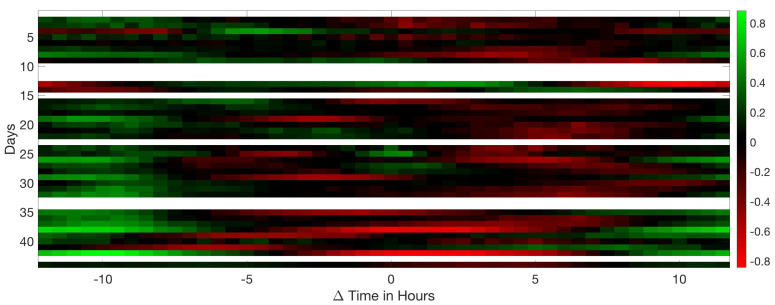
Results of the circular correlation humidity—$L_{eq}$. Horizontal axis corresponds to the ΔTime measured in hours, evaluating the delay between humidity and $L_{eq}$. Vertical axis stands for the days of the cycle.

A similar pattern can be seen in Figure 18, an inverse correlation is detected between humidity and the $L_{eq}$. The lower the humidity, the higher the sound level generated by the animals. Too much moisture in the chicken house contributes to the clamping of the bed and to ammonia problems. The animals are more vocally active when humidity values decreases.

**Figure 19:**
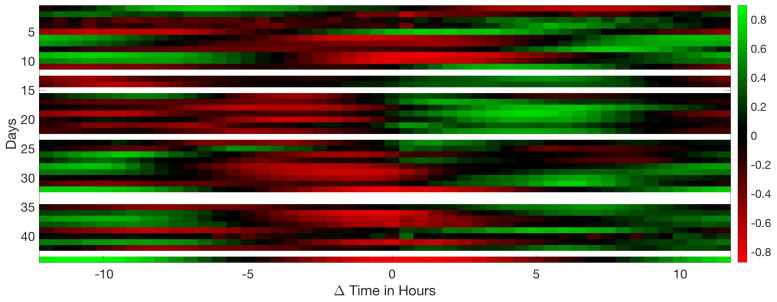
Results of the circular correlation temperature—humidity. Horizontal axis corresponds to the ΔTime measured in hours, evaluating the delay between temperature and humidity. Vertical axis stands for the days of the cycle.

Figure 19 shows a clearly inverse dependency between temperature and humidity. When temperature is at its maximum, the humidity is at its and vice-versa. As the air temperature rises, the amount of water that a given amount of air is able to retain increases. A 10 °C rise in temperature results in an approximate increase in air temperature halves the relative humidity.

**Figure 20:**
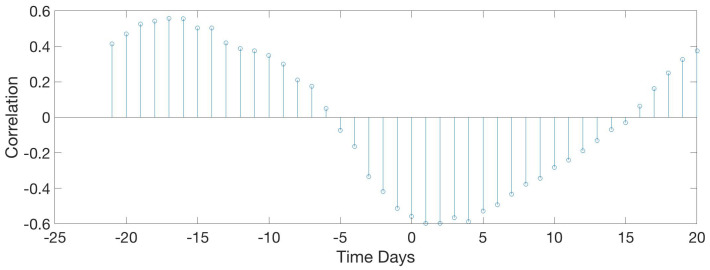
Results of the circular correlation food—max freq. Horizontal axis corresponds to the time (in days), evaluating the delay between the food intake and the maximum frequency detected.

Figure 20 shows an inverse relation between food intake and the mean max frequency vocalised by birds per day. When food intake is at a maximum, frequency is minimum. Max frequency is delayed two days from food. High frequency indicates high-pitched vocalisations that are related to stress, so they eat more when they are more relaxed.

**Figure 21:**
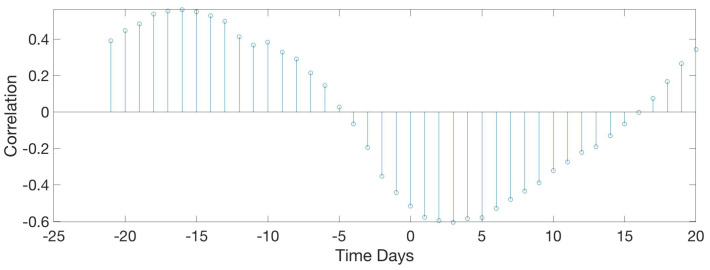
Results of the circular correlation water—max freq. Horizontal axis corresponds to the time (in days), evaluating the delay between the water intake and the maximum frequency detected.

Figure 21 follows a similar pattern as with the food consumption (Figure 20) an inverse relation between water intake and the mean maximum frequency vocalised by animals per day. When food intake is maximum, frequency is minimum. Maximum frequency is delayed by 2 days with respect to the water max values intake.

**Figure 22:**
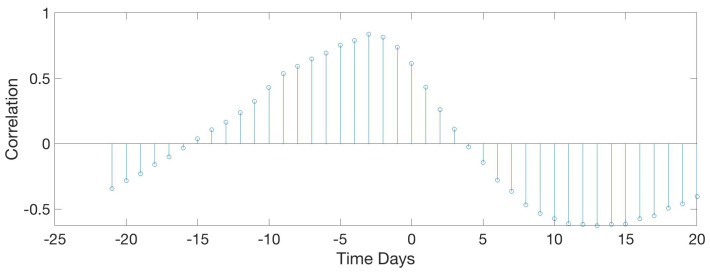
Results of the circular correlation food—weight. Horizontal axis corresponds to the time (in days), evaluating the delay between the food intake and the mean weight of the birds.

Figure 22 shows a correlation that does not depend on acoustic parameters but on the normal operation of the farm. It was an expected result, but noteworthy. Data indicates a direct dependency between food and weight with a significant correlation value. When food intake increases it also does the weight. Although weight is delayed 3 days with respect to the food intake values. This correlation corroborates the food-weight dependencies seen in the literature, transforming cereal protein to animal protein.

## 5. Discussion

In this work, a relation between farm indicators and acoustical metrics has been investigated. During the acoustic data acquisition the prevailing sound was the birds’ vocalisation, although some machinery sound data was also captured. The acoustical impact of the machinery increases the $L_{eq}$ and distorts the peak frequency of that acoustic fragment, therefore to avoid the analysis of this sounds as vocalisation a manual labelling process was implemented. For future, an automatic system could be implemented for a better and quick detection of the machinery sounds, especially with a previous training with the basic noise corresponding to the mechanics of the farm.

Once we had obtained the audio files with vocalisation predominance an analysis in terms of $L_{eq}$ and max frequency was performed. The peak frequency varies in function of birds day of life, a decreasing value is related with increasing age, a reduction of more than 1 kHz over the whole cycle. Furthermore, a variation is detected between light and dark lighting, with increased vocalisation during darkness. Frequency could be an indicator of birds days of life. The $L_{eq}$ is high during the light darkness of the farm.

Farm management practice depends on the following conditions: Season of the year, animal performance and the experience of the farmer. Air in the farm in winter reduces gases and also introduces cool air inside the house refreshing the ambient, meanwhile in summer ventilation introduces hot air and warms the farm. Farmers adjust the fans and heaters to maximise production in terms of economical costs and health. In the first week of production, animals are more susceptible to illness or sudden death, and they also require high ambient temperature to regulate internal temperature, 80% of death are premature in the first week. High values of farm management, high values of temperature, humidity, and CO_2_ reduces the vocal activity of the animal. Good farm management is relevant as reducing high values of temperature, humidity and CO_2_, increases the birds acoustic level. Bad management could lead to heat stress problem to the birds if the temperature index (in Fahrenheit) plus the humidity value sum exceeds 160. In winter, ventilating the farm reduces the CO_2_ but it also reduce the temperature of the house as the incoming air is cold. The amount of water kept in the air depends on the temperature, the higher the temperature, the higher the humidity.

Bird vocalisation represents the activity of the birds and also indicates distress calling caused by heat or cold stress, threat, pain, among others. Vocalisation can be detected through the peak frequency. An inverse relation has been found between the maximum frequency and food/water intake. The higher the food/water intake, the lower the peak frequency. A low peak frequency could indicate less stress and better welfare of the birds.

## 6. Conclusions and Future Work

Nowadays farm indicators (CO_2_, food, and water consumption, temperature, humidity) are used to monitor animal production and to maximise it, with a special focus on animal welfare. An acoustic recording of an entire production cycle (44 days) of broilers Ross 308 was performed to include in the metrics the data of animal acoustic vocalisation in terms of level and peak frequency. Special care has been considered to record the entirety of the production cycle so as to avoid losing any sound coming either from the birds or from machinery (or even from humans). This fact is relevant, due to the contribution of this work, which is to evaluate the relationship of the acoustic data with the farm management parameters (food and water intake, temperature, humidity), and also against the traditional indicators of deaths, evolution of the weight of the animals, and CO_2_ in the environment.

Acoustic data was captured with a cardioid microphone positioned in the centre of the farm and analysed to obtain the vocalisation indicators. All indicators, both acoustical and traditional farm ones, were analysed and compared, and several interesting relations were found that could enhance the evaluation of the animal welfare.

In this work we have obtained a couple of relevant preliminary conclusions. First, a relation between CO_2_ and humidity versus $L_{eq}$ shows an increasing of $L_{eq}$ when humidity or CO_2_ are lower. High values of CO_2_ and humidity reduce the acoustical activity of the birds, these high values generate discomfort of the birds and reduce animal welfare. Another relationship indicates that the higher the intake of food and water, the less frequency was found in the vocalisations A reduction in PF was related with quiet birds. Thus, animals consume more food and water when they are less stressed.

Further work will be focused on non-linear dependencies that all the gathered data can contain, after this first approach, using artificial intelligence algorithms. A deep study of the non-linear dependencies between variables will be performed. As we plan in some months to start another campaign in the framework of a new EuroStars project, several other considerations about the data collection and recording campaign design will be taken into account. Machinery noise in the farm should be exhaustively studied, and for this purpose, the labelling of any farm machine sounds will be conducted on the basis of a recording campaign without animals. Machinery noise can bias the results of the raw acoustic data analysis, and despite it being considered in this work when it modifies substantially the $L_{eq}$, mixtures of sounds among bird vocalisations and any mechanical noise should be at least identified.

Another issue to be improved upon is the number of acoustic sensors deployed in the farm. Multiple microphones enables multi-point recordings for having more spatially mapped levels and a better representation of the acoustic activity. For this new context, we plan to have at least three sensors in the same room of the farm in order to have redundancy in terms of acoustic measurements and possible metrics. In this sense, also the granularity of the data of the new environment will change the temporal windows to take into account for the study and the value chosen of 30 min may have to change to a more suitable time frame. Finally, an ISO standard for environmental noise recording will be required to be able to cross-site comparisons. Moreover, the results of this study will be compared to other productions cycles that will be carried on to determinate the stabilisation of the findings.

## Figures and Tables

**Table 1:** Average birds weight, measured in kg. The birds are weighted once per week, and the given value is the result of the average for several birds. Information collected from the farm management system.

Week Cycle	Mean (kg)
week 1	0.047
week 2	0.153
week 3	0.410
week 4	0.853
week 5	1.397

## Abbreviations

The following abbreviations are used in this manuscript:

FFT	Fast Fourier Transform
$L_{eq}$	Equivalent pressure level
CO_2_	Carbon dioxide
ppm	Parts per million
PCM	Pulse Code Modulation
SD	Secure Digital
H1	House One
H2	House Two
PF	Peak Frequency
MAX	Maximum
Eq	Equation

## References

[1] Meluzzi A., Sirri F. (2009). Welfare of broiler chickens. Ital. J. Anim. Sci..

[2] Panisello M. (2005). La patología y el medio ambiente en las granjas de broilers. Jornadas Profesionales de Avicultura de Carne.

[3] Mead G. (2004). Poultry Meat Processing and Quality.

[4] Petracci M., Mudalal S., Soglia F., Cavani C. (2015). Meat quality in fast-growing broiler chickens. World’s Poult. Sci. J..

[5] Gardiner E., Hunt J., Newberry R., Hall J. (1988). Relationships between age, body weight, and season of the year and the incidence of sudden death syndrome in male broiler chickens. Poult. Sci..

[6] Maxwell M., Robertson G. (1998). UK survey of broiler ascites and sudden death syndromes in 1993. Br. Poult. Sci..

[7] Ben Sassi N., Averós X., Estevez I. (2016). Technology and poultry welfare. Animals.

[8] Otu-Nyarko E. (2010). The Effect of Stress on the Vocalisations of Captive Poultry Populations. Ph.D. Thesis.

[9] Ginovart-Panisello G.J., Alsina-Pagès R.M. (2020). Preliminary Acoustic Analysis of Farm Management Noise and Its Impact on Broiler Welfare. Proceedings.

[10] Calvet S., Cambra-López M., Estelles F., Torres A. (2011). Characterization of gas emissions from a Mediterranean broiler farm. Poult. Sci..

[11] Pedersen S., Sällvik K. (2002). CIGR 4th Report of Working Group on Climatization of Animal Houses Heat and Moisture Production at Animal and House Levels.

[12] Norton T., Chen C., Larsen M.L.V., Berckmans D. (2019). Precision livestock farming: Building ‘digital representations’ to bring the animals closer to the farmer. Animal.

[13] Tefera M. (2012). Acoustic signals in domestic chicken (Gallus gallus): A tool for teaching veterinary ethology and implication for language learning. Ethiop. Vet. J..

[14] Zuberbühler K. (2009). Chapter 8 Survivor Signals: The Biology and Psychology of Animal Alarm Calling. Advances in the Study of Behavior.

[15] Clay Z., Smith C.L., Blumstein D.T. (2012). Food-associated vocalisations in mammals and birds: What do these calls really mean?. Anim. Behav..

[16] Delgado R. (2006). Sexual Selection in the Loud Calls of Male Primates: Signal Content and Function. Int. J. Primatol..

[17] Herborn K.A., McElligott A.G., Mitchell M.A., Sandilands V., Bradshaw B., Asher L. (2020). Spectral entropy of early-life distress calls as an iceberg indicator of chicken welfare. J. R. Soc. Interface.

[18] Fontana I., Tullo E., Scrase A., Butterworth A. (2016). Vocalisation sound pattern identification in young broiler chickens. Animal.

[19] Taylor A.M., Reby D. (2010). The contribution of source–filter theory to mammal vocal communication research. J. Zool..

[20] Manteuffel G., Puppe B., Schön P.C. (2004). Vocalization of farm animals as a measure of welfare. Appl. Anim. Behav. Sci..

[21] Fedurek P., Zuberbühler K., Dahl C.D. (2016). Sequential information in a great ape utterance. Sci. Rep..

[22] Briefer E., Comber S. (2012). Vocal expression of emotions in mammals: Mechanisms of production and evidence. J. Zool..

[23] Frewer L., Kole A., Kroon S., Lauwere C. (2005). Consumer Attitudes Towards the Development of Animal-Friendly Husbandry Systems. J. Agric. Environ. Ethics.

[24] Clark B., Stewart G.B., Panzone L.A., Kyriazakis I., Frewer L.J. (2017). Citizens, consumers and farm animal welfare: A meta-analysis of willingness-to-pay studies. Food Policy.

[25] Veissier I., Butterworth A., Bock B., Roe E. (2008). European approaches to ensure good animal welfare. Appl. Anim. Behav. Sci..

[26] Starke P. (2017). Comparative welfare state politics. Public Adm..

[27] Michael P., Mcloughlin R.S., McElligott A.G. (2019). Automated bioacoustics: Methods in ecology and conservation and their potential for animal welfare monitoring. J. R. Soc. Interface.

[28] Prunier A., Mounier L., Le Neindre P., Leterrier C., Mormède P., Paulmier V., Prunet P., Terlouw C., Guatteo R. (2013). Identifying and monitoring pain in farm animals: A review. Animal.

[29] Vandermeulen J., Bahr C., Tullo E., Fontana I., Ott S., Kashiha M., Guarino M., Moons C., Tuyttens F., Niewold T. (2015). Discerning pig screams in production environments. PLoS ONE.

[30] Watts J., Stookey J. (2000). Vocal behaviour in cattle: The animal’s commentary on its biological processes and welfare. Appl. Anim. Behav. Sci..

[31] Bishop J.C., Falzon G., Trotter M., Kwan P., Meek P.D. (2019). Livestock vocalisation classification in farm soundscapes. Comput. Electron. Agric..

[32] Whitaker B.M., Carroll B.T., Daley W., Anderson D.V. Sparse decomposition of audio spectrograms for automated disease detection in chickens. Proceedings of the 2014 IEEE Global Conference on Signal and Information Processing (GlobalSIP).

[33] Carpentier L., Vranken E., Berckmans D., Paeshuyse J., Norton T. (2019). Development of sound-based poultry health monitoring tool for automated sneeze detection. Comput. Electron. Agric..

[34] Lee J., Noh B., Jang S., Park D., Chung Y., Chang H.H. (2015). Stress detection and classification of laying hens by sound analysis. Asian-Australas J. Anim. Sci..

[35] Abdel-Kafy E.S.M., Ibraheim S.E., Finzi A., Youssef S.F., Behiry F.M., Provolo G. (2020). Sound Analysis to Predict the Growth of Turkeys. Animals.

[36] Du X., Carpentier L., Teng G., Liu M., Wang C., Norton T. (2020). Assessment of laying hens’ thermal comfort using sound technology. Sensors.

[37] Moura D.J.D., Nääs I.D.A., Alves E.C.D.S., Carvalho T.M.R.D., Vale M.M.D., Lima K.A.O.D. (2008). Noise analysis to evaluate chick thermal comfort. Sci. Agric..

[38] Aydin A., Bahr C., Viazzi S., Exadaktylos V., Buyse J., Berckmans D. (2014). A novel method to automatically measure the feed intake of broiler chickens by sound technology. Comput. Electron. Agric..

[39] Rowe E., Dawkins M.S., Gebhardt-Henrich S.G. (2019). A Systematic Review of Precision Livestock Farming in the Poultry Sector: Is Technology Focussed on Improving Bird Welfare?. Animals.

[40] Rios H.V., Waquil P.D., de Carvalho P.S., Norton T. (2020). How Are Information Technologies Addressing Broiler Welfare? A Systematic Review Based on the Welfare Quality^®^ Assessment. Sustainability.

[41] Astill J., Dara R.A., Fraser E.D.G., Roberts B., Sharif S. (2020). Smart poultry management: Smart sensors, big data, and the internet of things. Comput. Electron. Agric..

[42] Skomorucha I., Muchacka R., Sosnówka-Czajka E., Herbut E. (2009). Response of broiler chickens from three genetic groups to different stocking densities. Ann. Anim. Sci..

[43] Bernard P. (1975). SEL: When? Why? How?.

[44] Zoom Corporation (2014). H5 Handy Recorder—Operation Manual.

[45] Behringer (2011). Ultravoice XM1800S Technical Specifications.

[46] Fontana I., Tullo E., Butterworth A., Guarino M. (2015). An innovative approach to predict the growth in intensive poultry farming. Comput. Electron. Agric..

[47] Fontana I., Tullo E., Carpentier L., Berckmans D., Butterworth A., Vranken E., Norton T., Berckmans D., Guarino M. (2017). Sound analysis to model weight of broiler chickens. Poult. Sci..

[48] Aviagen (2017). The Procedure for Individually Weighing Broilers from 21–28 Days Onwards.

[49] MATLAB (2020). 9.8.0.1359463 (R2020) Update 1.

[50] Namba S., Kuwano S. (1984). Psychological study on Leq as a measure of loudness of various kinds of noises. J. Acoust. Soc. Jpn..

[51] ISO Central Secretary (1982). Acoustics—Description and Measurement of Environmental Noise—Part 1: Basic Quantities and Procedures.

[52] Podder P., Khan T.Z., Khan M.H., Rahman M.M. (2014). Comparative performance analysis of hamming, hanning and blackman window. Int. J. Comput. Appl..

[53] Cooley J.W., Tukey J.W. (1965). An algorithm for the machine calculation of complex Fourier series. Math. Comput..

[54] Glover R.P. (1940). A review of cardioid type unidirectional microphones. J. Acoust. Soc. Am..

[55] Oppenheim A.V. (1999). Discrete-Time Signal Processing.

